# Supplementary material for: A Corrosion Sensor for Monitoring the Early-Stage Environmental Corrosion of A36 Carbon Steel
Source: Materials (Basel). 2014 Aug 8;7(8):5746–60. doi: 10.3390/ma7085746 (PMC5456204; doi:10.3390/ma7085746)
Supplement: Supplementary File 1 [file materials-07-05746-s001.pdf]

Supporting Information

Figure S1. A corrosion sensor and the sensor system installed on a steel bridge over Mississippi River.

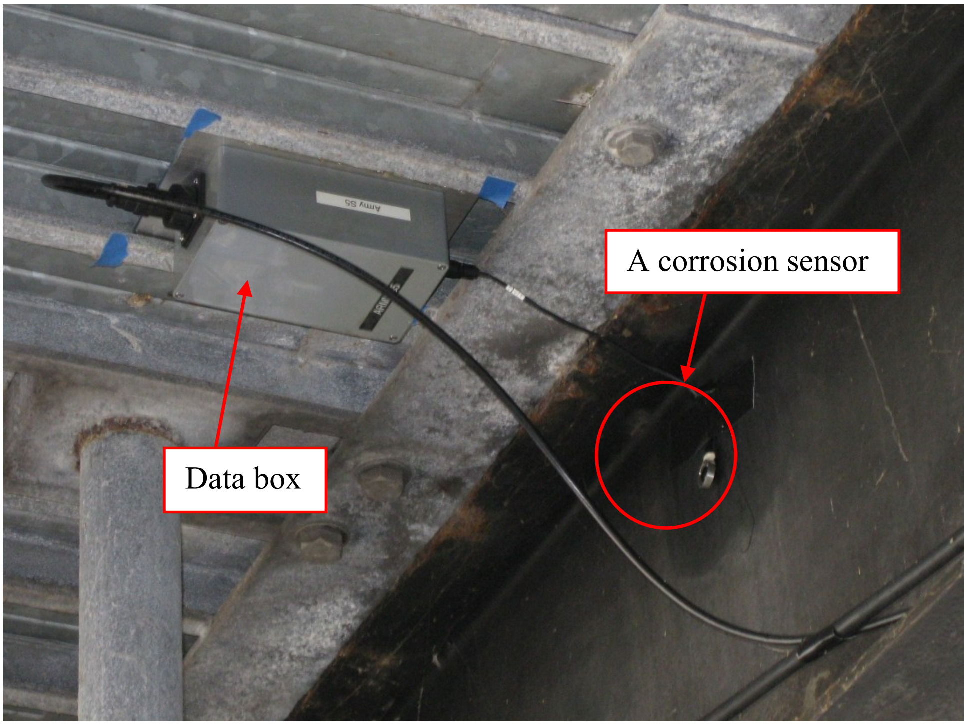

Figure S2. The corrosion data acquisition and monitoring system.

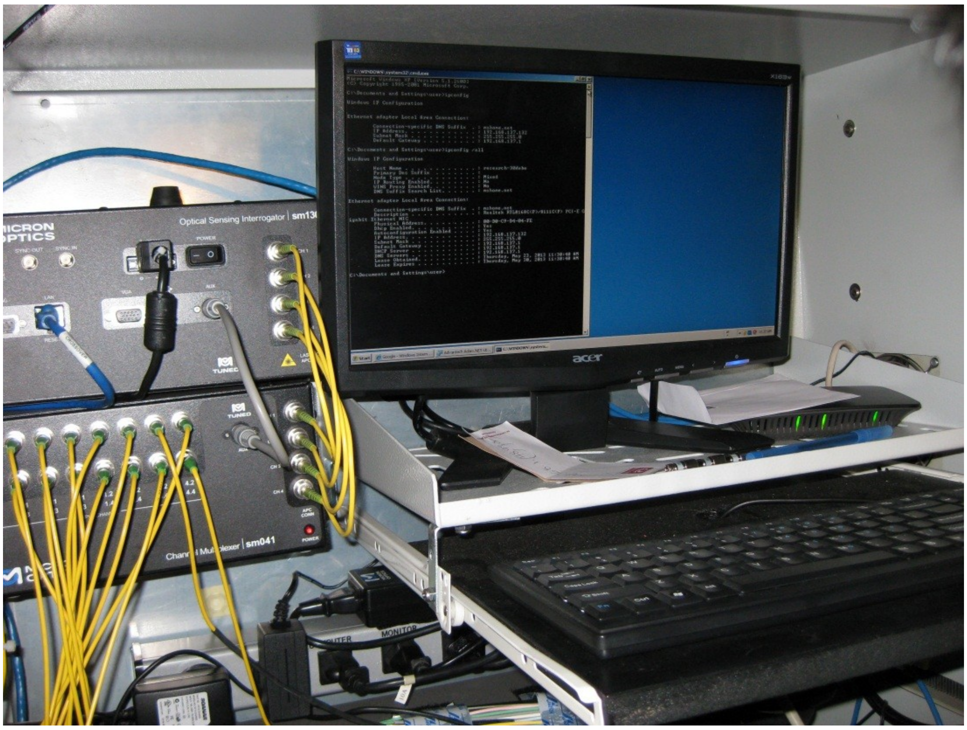

When fringe field is considered, Heerens [1] proposed the following equation to calculate the approximate value of capacitance.

$$C = \frac{4\varepsilon_0\varepsilon(b-a)}{\pi}\sum_{n=1}^{\infty}(-1)^n\,[1-\cos(nz)]^2[n^2W_n(r_1,r_2)]^{-1} \tag{S1}$$

where C = sensor's capacitance (pF); ε = dielectric constant of the material(s) between the A36 steel rod and the 316 stainless steel ring; a = radius of the A36 steel rod (m); b = inner radius of the 316 stainless steel ring (m); h = height of the cylinder sensor (m); $z = \pi h/(b-a)$; $r_1 = \pi a/(b-a)$; $r_2 = \pi b/(b-a)$.

$W_n(r_1,r_2) = I_0(nr_1)K_0(nr_2) - K_0(nr_1)I_0(nr_2)$, in which $I_0(u)$ and $K_0(u)$ are the modified Bessel functions of the first and second kind and of zero order which for an argument u, respectively.

Similar to Equation (13) in the paper, a much more complicated equation of multi-material capacitance in-series with considering fringing effect can be derived. In agreement with Equation (13), the capacitance with fringe effect shows that the capacitance change is more sensitive to ε, the dielectric constant of the material(s) (*i.e.*, rust and air) between the A36 steel rod and the 316 stainless steel ring than the change of a, the radius of the A36 steel rod.

Reference

1. Heerens, W.C. Application of capacitance techniques in sensor design. *J. Phys. E: Sci. Instrum.* **1986**, *19*, 897–906.
